# Effect of Process Parameters on Properties of Cold-Sprayed Zn–Al Composite Coatings

**DOI:** 10.3390/ma15197007

**Published:** 2022-10-09

**Authors:** Naijiang Wang, Chengxin Liu, Yangang Wang, Hao Chen, Xingrong Chu, Jun Gao

**Affiliations:** 1 Associated Engineering Research Center of Mechanics and Mechatronic Equipment, Shandong University, Weihai 264209, China; 2 Inner Mongolia Power (Group) Co., Ltd., Inner Mongolia Power Research Institute Branch, Hohhot 010020, China; 3 Inner Mongolia Enterprise Key Laboratory of High Voltage and Insulation Technology, Hohhot 010020, China

**Keywords:** cold spraying, Al–Zn coating, corrosion properties, microhardness, porosity

## Abstract

Al–Zn composite coating can provide effective cathodic protection for E235 steel. This study aims to obtain the Al–Zn composite coating with the best anti-corrosion performance by optimizing the spraying temperature, spraying distance and powder-feeding motor speed. The Al and Zn powders were analyzed by scanning electron microscope (SEM), and the microstructure of the coatings prepared by different process parameters was observed by optical microscope. The mechanical and anticorrosive properties of the coating were evaluated using hardness, porosity, thickness and electrochemical tests. According to the experimental results, when the spraying temperature, spraying distance and powder-feeding motor speed were 500 °C, 27 mm and 1.5 r/min, respectively, the hardness of the coating was 67 HV, the porosity was 0.57% and the thickness was 0.588 mm. The EIS test results show that the coating has the maximum polarization resistance, and therefore the coating has good corrosion resistance at this parameter.

## 1. Introduction

E235 steel is a kind of ordinary carbon structural steel with good mechanical performance, plasticity, toughness and strength, and is a commonly used plate at present. However, it is easy to produce surface cracks, corrosion and other defects in the working process. In the long run, equipment made of E235 may not operate normally, stably and safely [1]. Therefore, it is urgent to carry out proper surface treatment for protection and repair [2]. Common surface treatment methods include electroplating, electroless plating and thermal spraying techniques such as supersonic flame spraying and plasma spraying. However, the above technologies have a complex operation process, high production cost, and thin coating thickness with low protection ability [3]. Due to the high spraying temperature of thermal spraying technology, easily oxidized powder or substrate will be oxidized, reducing the performance [4]. As a protective coating preparation technology, cold-spraying technology attracts wide attention because of its low working temperature, which could retain the original properties of the powder and substrate, and could quickly prepare coatings of a certain thickness [5].

The deposition of cold-spray particles is a mechanical process. Particles with high kinetic energy and high velocity (typically 300 to 1200 m/s) cause interactions between the deposited surface and individual particles, resulting in a coating buildup. The deposition of the coating depends on the plastic deformation of the solid particles rather than the heat input [6,7]. For a particular material, the degree of plastic deformation depends on the particle velocity [8]. Based on the above discussion, cold-spraying technology is suitable for heat-sensitive materials [5,9], and appropriate process parameters can improve the particle deposition effect and coating performance [10].

Al and Zn coatings have good corrosion resistance and could provide effective protection for the substrate [11,12,13]. Maledi [14] uses cold-spraying technology to prepare a zinc coating on a steel surface, and discusses the relationship between process parameters and coating properties and structure. When the temperature is 500 °C and the pressure is 0.7 MPa, the hardness of the coating is the highest. Zhang [15] successfully deposits three kinds of dense Al-based coatings by low-pressure cold spraying, and these coatings could provide excellent long-term anticorrosive protection for the substrate. Zhang [16] prepares Al2024-Al_2_O_3_ coating with different Al_2_O_3_ content onto a 2024-T3 alloy substrate, and the experimental results show that the corrosion resistance of the coating is the best when the mass fraction of Al_2_O_3_ is 20%. Kumar [17] studies the correlation between SiC particle fraction and the mechanical properties of cold-sprayed Al-SiC MMC coating. Wang [18] prepares SiC particle-reinforced Al5056 alloy coating and Al5056 substrate composite coating with a particle size of 20μm and high spraying efficiency and the best corrosion resistance. Xie [19] prepares a pure Zn coating on an AZ31b substrate by cold spraying and it is characterized by electrochemical performance. The results show that a Zn coating could protect an AZ31b substrate to a great extent. Legoux [20] studies the effect of substrate temperature on the formation mechanism of low-melting-point coatings (Al, Zn and Sn). It is found that the surface temperature has a negative effect on the deposition efficiency of the Zn coating, and a high driving gas temperature will reduce the hardness of the Zn coating. Wu [21] uses cold-spraying technology to prepare a Zn-G/Al coating on carbon steel, and the results show that the coating has cathodic protection abilities on steel.

In this study, a Zn–Al composite coating is prepared on E235 steel using cold-spraying technology. The aim is to optimize the spraying temperature, spraying distance and feeding motor speed, and improve the corrosion performance of the coating. The microstructure of the coating is analyzed by optical microscope. The corrosion resistance and mechanical properties of the coating are evaluated by electrochemical and hardness tests. Relevant experiments have been carried out without any systematic study on the effect of the process parameters on the microstructure and properties of cold-sprayed Zn–Al coating. Therefore, based on P800Q2-Ⅱ automatic portable cold-spraying equipment, this paper prepares a cold-sprayed Zn–Al composite coating on an E235 substrate. By optimizing different process parameters, the effects of these parameters on the coating properties are studied. It provides theoretical guidance for preparing cold-spray Zn–Al coating.

## 2. Materials and Methods

### 2.1. Experimental Materials

**Figure 1:**
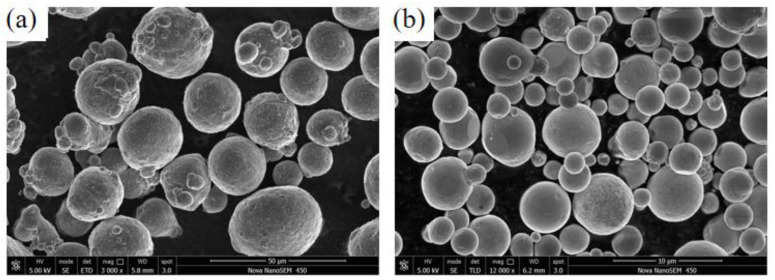
Particle morphology diagram: (**a**) aluminum particles, (**b**) zinc particles.

The chemical elements of aluminum zinc powder are shown in Table 1. The morphology of the powder materials used in the experiments was observed using a scanning electron microscope (SEM, Nova Nano SEM450, FEI Sirion, Hillsboro, OR, USA), as shown in Figure 1. As can be seen from Figure 1, the particle sizes of the aluminum powder and zinc powder were 30–50 μm and 5–30 μm, respectively. The E235 plate was 100 mm × 60 mm × 3 mm, and the chemical composition of the plate is shown in Table 2.

### 2.2. Experimental Methods

#### 2.2.1. Pretreatment of Powder and Substrate

First, the aluminum powder and zinc powder with a mass ratio of 3:1 were weighed. Then, the mixed powder was dried for 2 h in a vacuum-drying oven at 90 °C to remove the moisture and to improve the fluidity of the powder and facilitate mechanical mixing. The dried powder was poured into a 3D moving mixer for mechanical mixing for 1 h to ensure an even mixture. The substrate surface of E235 was treated with sandpaper and cleaned with alcohol.

#### 2.2.2. Coating Preparation

**Figure 2:**
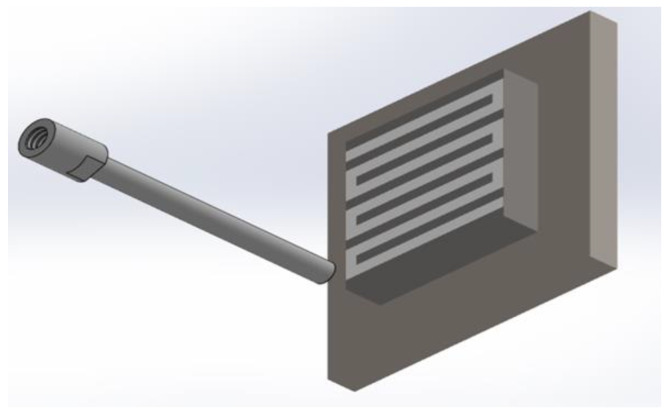
Trajectory of the spray gun.

The coating preparation experiments mainly included optimization experiments of spraying temperature, spraying distance and powder-feeding motor speed. The specific spraying parameters are shown in Table 3. All spraying experiments were done using P800Q2-Ⅱ automatic portable cold-spraying equipment. N_2_ was used as the compressed gas, and the spraying pressure was 1.9 MPa. The corresponding coating samples were obtained by spraying the substrate four times. The spray track adopted the shape shown in Figure 2.

#### 2.2.3. Performance Detection Method

**Figure 3:**
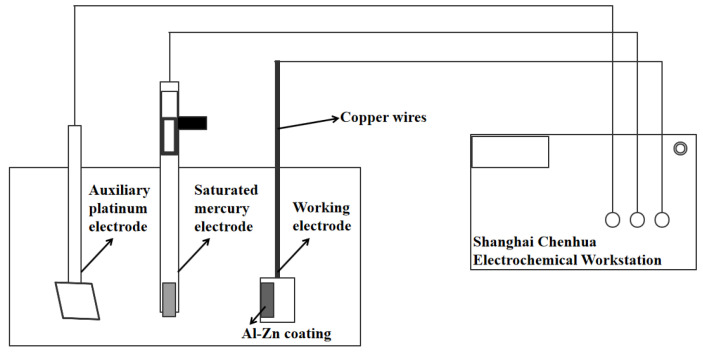
Schematic diagram of coated electrode and electrochemical test system.

The surface and cross-section of the coating were observed with a 3D laser scanning confocal microscope (VK-X1000; KEYENCE, Osaka, Japan), and the surface roughness Ra and thickness of the coating were measured. ImageJ was used to characterize the porosity of the coating cross-section. The hardness of the coating was measured by a MHV-1000Z Vickers microhardness tester with a load of 50 g and a holding time of 10 s. Five points on the cross-section of the coating were selected for the hardness test, and the average value was taken as the coating hardness. In order to test the corrosion resistance of the coating sample, the electrochemical performance of the coating sample was tested. All electrochemical experiments were performed based on the CHI660E electrochemical workstation (Shanghai Chenhua Instrument Co., Ltd., Shanghai, China). A cold-sprayed Al–Zn coating sample was used as the working electrode, with an area of 5 mm × 5 mm. The reference electrode was saturated calomel electrode (SCE) and the auxiliary electrode was a 10 mm × 10 mm platinum sheet. The working solution used for the test was 3.5 wt.% NaCl solution. Impedance spectroscopy (EIS) was performed in the electrochemical experiment, and the frequency range was 10 mHz~100 KHz. A diagram of the test system is shown in Figure 3. The OCP test was performed before all EIS experiments, and the EIS test was performed according to the open-circuit potential obtained from the OCP test.

## 3. Results and Analysis

### 3.1. Microstructure

**Figure 4:**
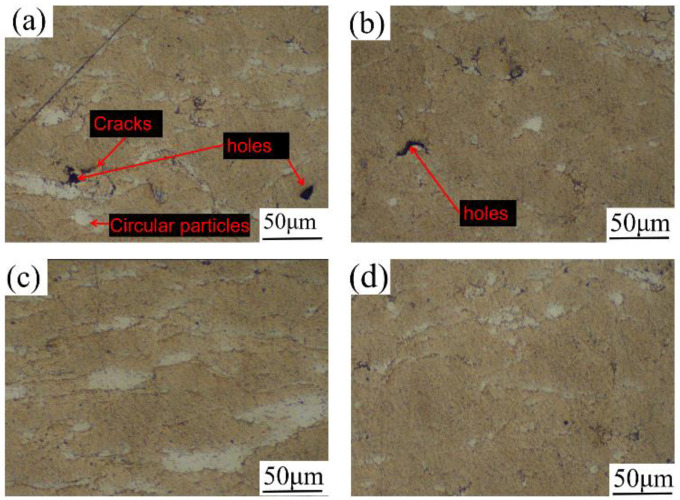
Microstructure of coating sections at different spraying temperatures: (**a**) 400 °C, (**b**) 450 °C, (**c**) 500 °C, (**d**) 550 °C.

The cross-section microscopic morphology of the coating at different spraying temperatures is shown in Figure 4. The coating mainly included gray-yellow and white areas, and the gray-yellow area was obviously more than the white area. Based on the deformation ability of the particles and the composition ratio of the coating material, the gray-yellow area was the Al element and the white area was the Zn element. According to Figure 4a, when the spraying temperature was 400 °C, there were nearly spherical Zn particles in the coating, and the particle deformation degree was small. When the spraying temperature was 500 °C, the particle deformation was sufficient and the bonding was tight with few defects, as shown in Figure 4c. As seen in Figure 4d, when the spraying temperature was 550 °C, the number of defects increased compared with those at 500 °C, and the particle deformation degree weakened. The reason for this phenomenon may be that at this temperature, the particle velocity exceeded the critical velocity and eroded the coating [22]. ImageJ was used to calculate the porosity of the coating section. The porosity of the coating was the highest at 400 °C, which was 1.41%. The porosity of the coating at 450 °C, 500 °C and 550 °C was 0.88%, 0.71% and 0.75%, respectively, which was consistent with the microscopic observation results.

**Figure 5:**
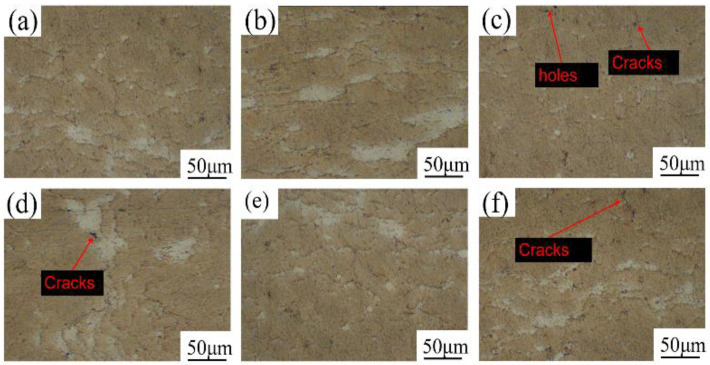
Microstructure of coating sections at different spraying distances: (**a**) 18 mm, (**b**) 20 mm, (**c**) 22 mm, (**d**) 25 mm, (**e**) 27 mm, (**f**) 30 mm.

The microscopic morphology of the coating cross-section at different spraying distances is shown in Figure 5. The coating also consisted of a large amount of Al in the gray-yellow region and a small amount of Zn in the white region. When the spraying distance was 18–30 mm, the number of defects in the coating section decreased first, and then increased. This was due to the superposition effect of the resistance wave and carrier gas on the particle acceleration [22]. When the spraying distance was 27 mm, the coating defects were fewer, there were almost no round particles, and the particle deformation degree was more severe. The results showed that the porosity of the coating was 0.79%, 0.71%, 0.67%, 0.63%, 0.57% and 0.73%, respectively, with the increase in spraying distance. When the spraying distance was 18 mm, the porosity of the coating was the highest, which was mainly because the spraying distance was too small, the particle acceleration effect was not obvious, and the resistance wave of the substrate surface had a great influence on the particles. However, when the spraying distance was 27 mm, the porosity of the coating was the lowest when the spraying distance was appropriate.

**Figure 6:**
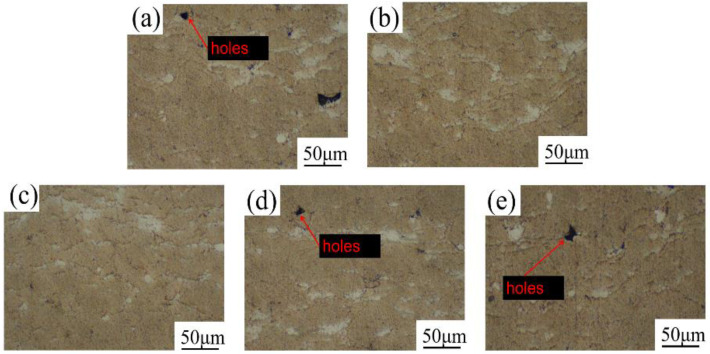
Microstructure of coating sections at different spraying speeds of powder-feeding motor: (**a**) 1.0 r/min, (**b**) 1.2 r/min, (**c**) 1.5 r/min, (**d**) 1.7 r/min, (**e**) 2.0 r/min.

The cross-section microscopic morphology of the coating at different spraying speeds of the powder-feeding motor is shown in Figure 6. The coating also consisted mostly of Al in the gray-yellow areas and a small amount of Zn in the white areas. With the increase in the feeding motor speed, the number of coating cracks decreased first, and then increased. When the motor speed was 1.5 r/min, there were fewer coating defects and the bonding was the closest. The calculation results of the porosity of the coating section showed that the porosity was 0.70%, 0.64%, 0.57%, 0.68% and 0.69%, respectively, with the increase in the feeding motor speed. The results showed that a motor speed that is too high or too low leads to an increase in the number of coating defects. The main reason was that when the speed of the powder motor was small, the number of particles in the airflow was small, and the tamping effect of particles on the coating was not obvious, resulting in poor coating performance. When the speed of the powder-feeding motor was too large, the carrier gas had a limited effect on particle acceleration, which led to the decrease in particle kinetic energy, the enhancement of particle interaction, and the deterioration of coating performance.

### 3.2. Coating Thickness and Roughness Test

**Figure 7:**
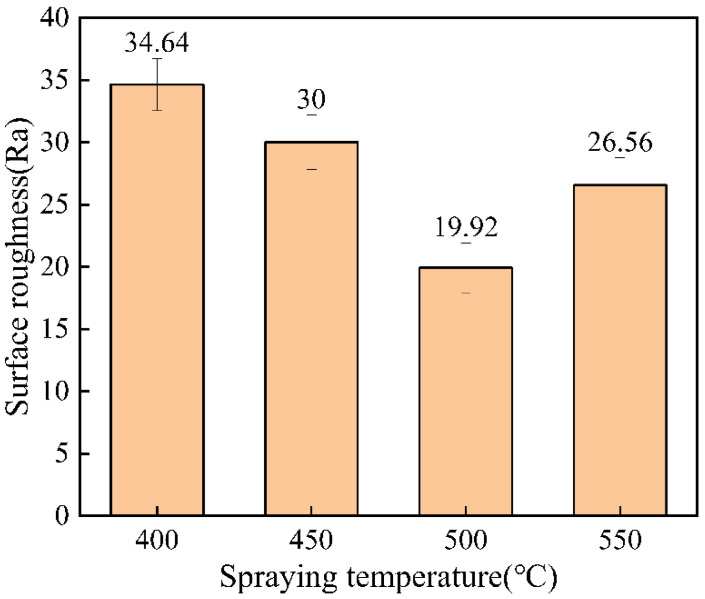
Surface roughness of coating at different spraying temperatures.

**Figure 8:**
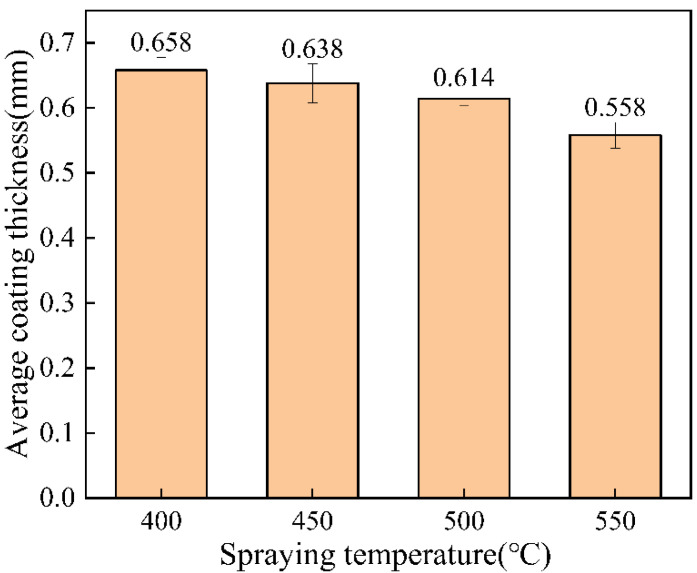
Average coating thickness at different spraying temperatures.

Surface roughness could reflect the deformation of cold-spraying particles to a certain extent [23]. The surface roughness of the coating is shown in Figure 7. When the spraying temperature was 500 °C, the surface undulation of the coating was the smallest, and when the spraying temperature was 400 °C, the surface of the coating was relatively rough. The average coating thickness at different spraying temperatures is shown in Figure 8. According to Figure 8, the coating thickness decreased with the increase in temperature, and the coating thickness changed little between 400 °C and 500 °C. When the spraying temperature was 550 °C, the coating thickness was small. This phenomenon occurred mainly because the particle velocity increased with the increase in temperature. Erosion occurred when the particle velocity exceeded the critical velocity, resulting in a relatively thin coating thickness [22,24,25].

**Figure 9:**
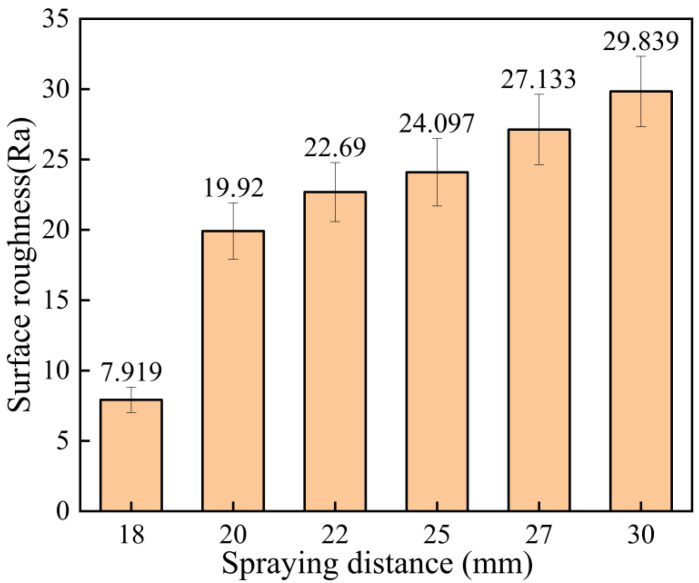
Surface roughness of the coating at different spraying distances.

**Figure 10:**
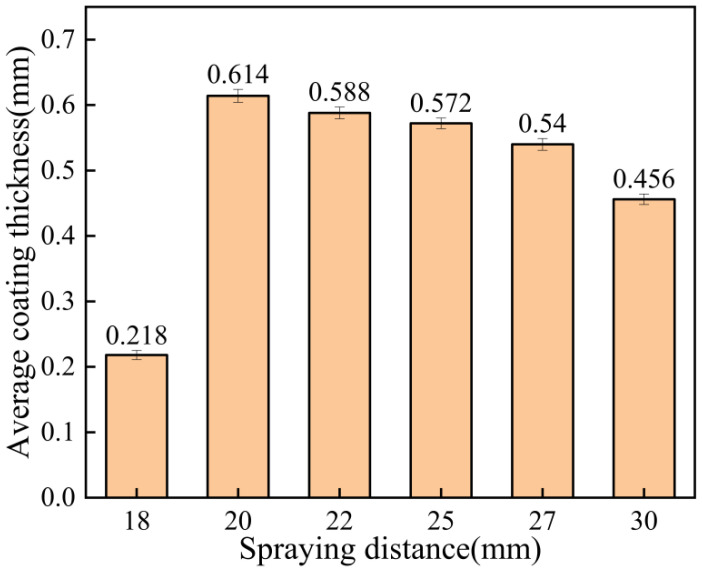
Average coating thickness at different spraying distances.

The surface roughness of the coating at different spraying distances is shown in Figure 9. As can be seen from Figure 9, the coating surface formed when the spraying distance was 18 mm was the most delicate, while the coating surface was relatively rough when the spraying distance was 30 mm. The average coating thickness at different spraying distances is shown in Figure 10. It can be seen that the coating thickness was the thinnest when the spraying distance was 18 mm, which means that under certain process parameters, when the coating is deposited to a certain thickness, it becomes more difficult for the subsequent particles to be deposited. That is, the deposition rate and erosion rate of the coating reached the equilibrium stage [26]. However, subsequent particles will continue to forge the deposited coating, producing an effect of polishing the coating surface, so the resulting coating is thin with low roughness. When the spraying distance was 20 mm, the maximum thickness was 0.614 mm, and at 30 mm, the thickness was 0.456 mm. When the spraying distance was 20–30 mm, the acceleration effect of the carrier gas on the particles and the comprehensive effect of the resistance wave on the surface of the substrate gradually decreased the kinetic energy of the particles, so the thickness decreased gradually.

**Figure 11:**
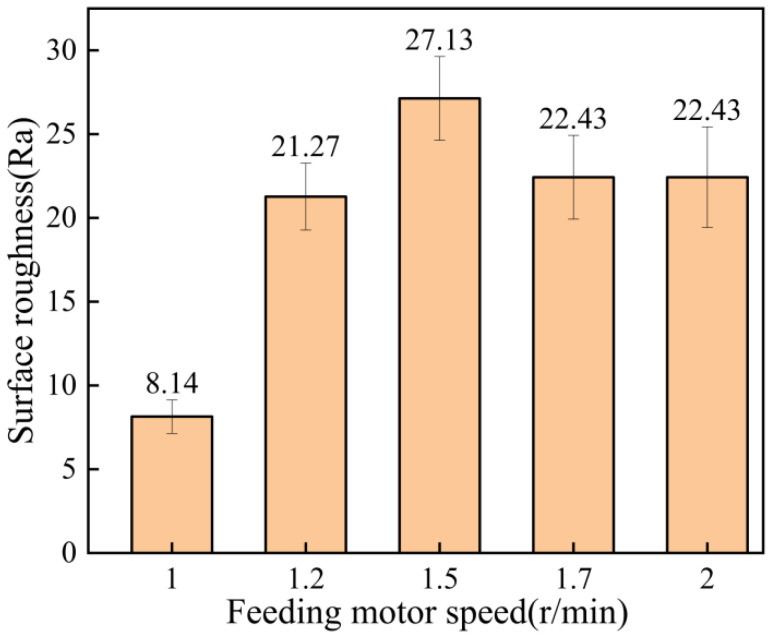
Surface roughness of coating at different feeding motor speeds.

**Figure 12:**
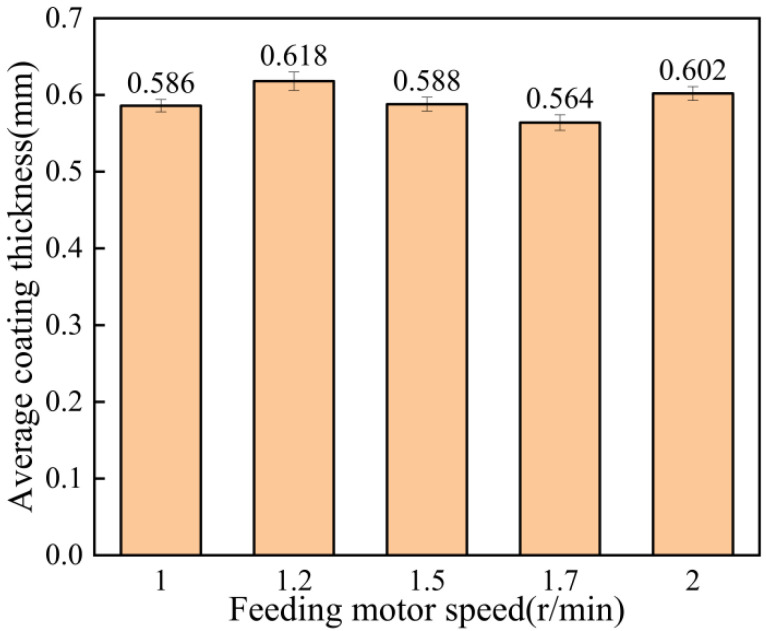
Average coating thickness at different feeding motor speeds.

The surface roughness of the coatings at different feeding motor speeds is shown in Figure 11. As can be seen from the figure, the coating surface formed when the speed of the powder-feeding motor was 1.0 r/min, which was the most delicate. However, the coating formed when the speed was 1.5 r/min was the coarsest. Figure 12 shows the average thickness of the coating at different feeding motor speeds. It can be seen from the figure that in a certain range, the speed of the powder-feeding motor had little influence on the coating thickness, and the coating thickness was about 0.6 mm. The minimum thickness of the coating was 0.564 mm, but the maximum thickness of the coating was 0.618 mm at 1.2 r/min. When the speed of the powder-feeding motor was 1.0 r/min, the coating thickness was small. Because the slower the speed of the powder-feeding motor, the smaller the powder content in the spray gun per unit time, the worse the subsequent particle compaction effect, the longer the time taken to reach the activation cycle, and the smaller the coating thickness [27,28].

### 3.3. Microhardness

**Figure 13:**
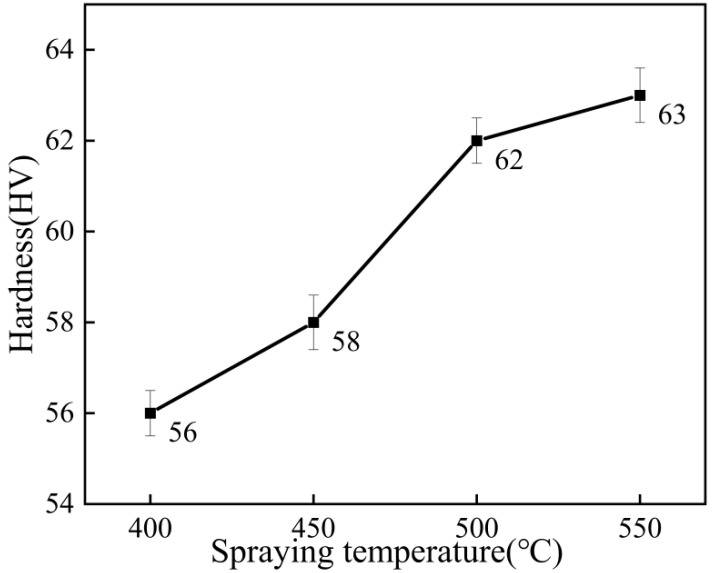
Microhardness of the coating at different spraying temperatures.

Figure 13 shows the microhardness test results of Al–Zn composite coating samples at different spraying temperatures. As can be seen from the figure, the microhardness of the coating increased from 56 HV to 63 HV with the increase in the spraying temperature. As can be seen from Figure 4 of the coating section, when the spraying temperature was 400 °C and 450 °C, the density of the coating was low, the particles were not tightly bonded, and there were defects and insufficiently deformed spherical Zn particles in the coating, so the microhardness was low. When the spraying temperature was 500 °C, the particle acceleration effect was obvious, and the coating density was increased, so the average microhardness of the coating was higher at this temperature.

**Figure 14:**
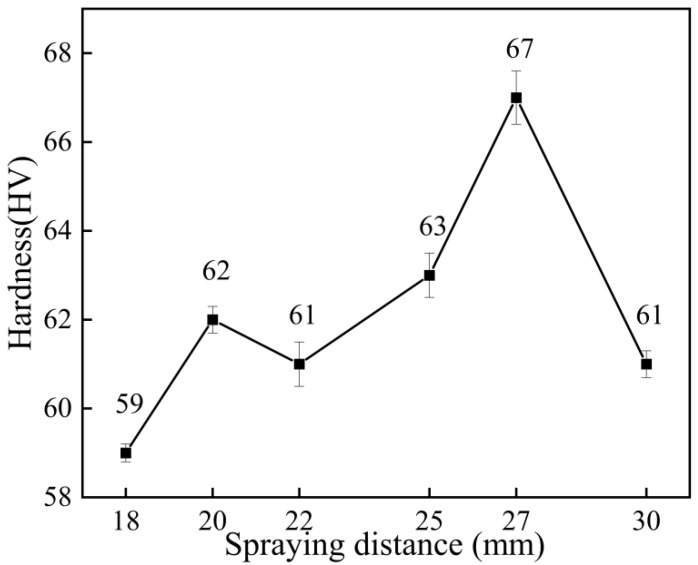
Microhardness of the coating at different spraying distances.

Figure 14 shows the microhardness test results of the coating at different spraying distances. As can be seen from the figure, the microhardness of the coating increased first and then decreased with the increase in the spraying distance. The maximum microhardness of the coating was 67 HV when the spraying distance was 27 mm, and the minimum was 59 HV when the spraying distance was 18 mm. The reason for the difference in the coating’s hardness is that the change of distance leads to changes in the shockwave on the surface of the substrate, and then this affects the particle velocity, leading to the different hardening effects of the coating [28]. It might also be because the particle acceleration effect was not obvious when the spraying distance was small, but the particle “scattering phenomenon” was more serious when the spraying distance was greater [29]. Therefore, when the spraying distance was 18 mm, the particle acceleration effect was not obvious, and when the spraying distance was 30 mm, the particle “scattering phenomenon” was more prominent. When the spraying distance was 27 mm, the particle deformation was sufficient, the coating was compact, and the work hardening effect was obvious.

**Figure 15:**
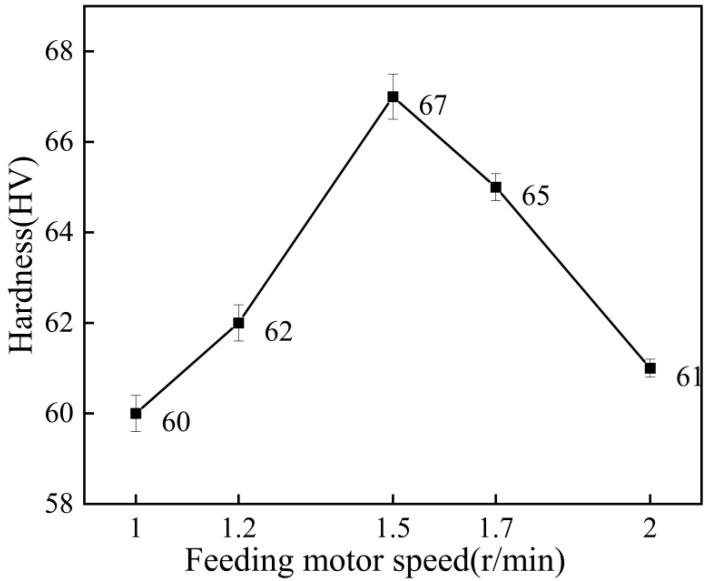
Microhardness of the coating at different feeding motor speeds.

Figure 15 shows the microhardness test results of the coating at different feeding motor speeds. As can be seen from the figure, with the increase in the speed of the powder-feeding motor, the microhardness of the coating on the whole showed a trend of first increasing and then decreasing. The maximum microhardness of the coating was 67 HV when the motor speed was 1.5 r/min, and 60 HV when the motor speed was 1.0 r/min. When the feeding motor speed was 2 r/min, the microhardness of the coating decreased. The reason for this phenomenon was that when the feeding motor speed was too slow, the particles had a poor tamping effect on the coating, and the work hardening was not obvious. When the speed of the powder-feeding motor was too fast, the particles far from the axis of the spray gun increased. The particles farther away from the axis had lower velocities. This hardness difference occurs because the work hardening of the coating was reduced, as shown in Equation (1) [30].

When the feeding motor speed was 1.5 r/min, the particles could be fully accelerated, the interaction between particles was small, the subsequent particle tamping effect was obvious and the work-hardening effect was the most obvious, so the microhardness of the coating was the highest.

(1) $$\frac{v}{v_{m}}={\left(\frac{\delta }{y}\right)}^{-1/7}$$

### 3.4. Electrochemical Performance Test

**Figure 16:**
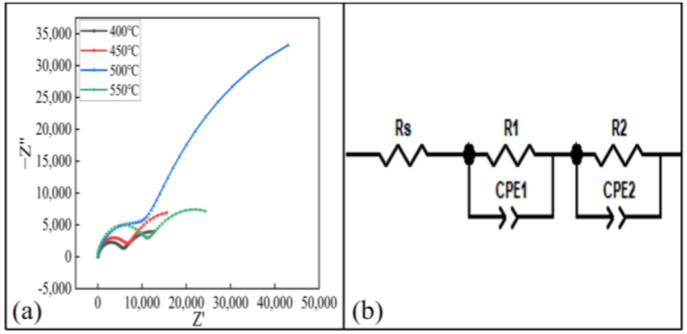
Electrochemical test results of the coating at different spraying temperatures: (**a**) Nyquist diagram, (**b**) equivalent circuit model diagram.

In order to study the corrosion behavior of the coating, the impedance spectrum of the coating was tested. The Nyquist of the coating at different spraying temperatures is shown in Figure 16a. The Nyquist diagram of each coating sample presented two semicircular shapes. It can be seen from the figure that the capacitance–reactance arc radius of the coating with a spraying temperature of 500 °C was the largest, so it had better barrier function [21]. The experimental EIS results were fitted by the equivalent circuit diagram shown in Figure 16b. Since the electrode system used in the experiment was not ideal, the capacitor element was replaced with a constant phase angle element (CPE) in the fitting process [16]. In the fitted circuit, R_s_, R_1_ and R_2_ were the solution resistance, the charge transfer resistance and the additional resistance of the corrosion part, respectively. The constant phase element CPE1 was in parallel with R_1_, corresponding to a high-frequency semicircle. R_2_ was in parallel with the constant phase angle original CPE2. Take the sum of R_1_ and R_2_ to represent the corrosion resistance R_e_ of the coating, that is, R_e_ = R_1_ + R_2_ [31]. The R_s_ and R_e_ of coatings sprayed at different spraying temperatures are shown in Table 4. It can be seen that the R_e_ value of the 500 °C coating was the largest, so the anticorrosion performance of the coating was the best when the spraying temperature was 500 °C.

**Figure 17:**
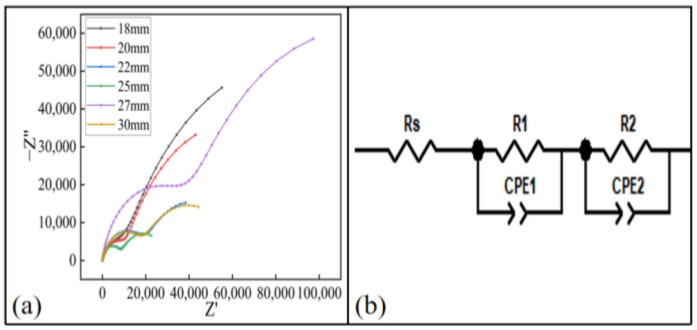
Electrochemical test results of the coating at different spraying distances: (**a**) Nyquist diagram, (**b**) equivalent circuit model diagram.

The Nyquist of the coating at different spraying distances is shown in Figure 17a. The Nyquist diagram of each coating sample presented two semicircular shapes. As can be seen from the figure, when the spraying distance was 27 mm, the capacitive arc radius of the coating was the largest, which had a better barrier function. This was because when the spraying distance was 27 mm, the particles were fully accelerated by the nozzle gas and were less affected by the shockwave on the substrate surface [32], and could impact the substrate at a high speed. Therefore, the anticorrosion performance was optimal. The EIS results were fitted using the equivalent circuit diagram shown in Figure 17b. the resistance R_s_ and R_e_ of the coating solution at different spraying distances are shown in Table 5. It can be seen that the R_e_ value of the coating was the largest at 27 mm, so the anticorrosion performance of the coating was the best.

**Figure 18:**
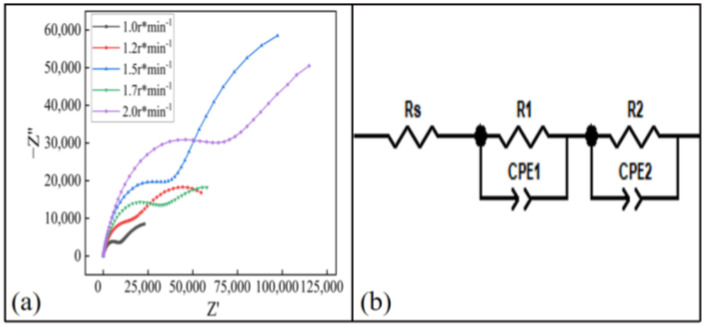
Electrochemical test results of the coating at different feeding motor speeds: (**a**) Nyquist diagram, (**b**) equivalent circuit model diagram.

The Nyquist of the coating at different feeding motor speeds is shown in Figure 18a. It can be seen from the figure that the capacitive arc radius of the coating was the largest when the speed of the powder-feeding motor was 1.5 r/min, which had a better barrier function. The experimental EIS results were still fitted using the equivalent circuit diagram shown in Figure 18b. The resistance R_s_ and R_e_ of the coating solution at different speeds of the powder-feeding motor are shown in Table 6. It can be seen that when the speed of the powder-feeding motor was 1.5 r/min, the R_e_ value of the coating was the largest, which is 196,320 Ω. Therefore, when the speed of the powder-feeding motor was 1.5 r/min, the anti-corrosion performance of the coating was the best.

## 4. Conclusions

In this study, an Al–Zn composite coating was prepared on the surface of E235 steel using cold-spraying technology. The Al–Zn coating with different process parameters was taken as the main research object, aiming to optimize the parameters to improve the corrosion performance of the coating. The main conclusions were as follows.

With the increase in the spraying temperature, spraying distance and feeding motor speed, the coating pores first decreased and then increased. The hardness and corrosion resistance of the coating increased first and then decreased. When the spraying temperature was 500 °C, the spraying distance was 27 mm and the feeding motor speed was 1.5/min, the coating structure was the densest and the corrosion resistance was the best.

When the spraying temperature was 500 °C, the spraying distance was 27 mm and the feeding motor speed was 1.5 r/min, the performance of the Al–Zn composite coating was the best. The hardness could be up to 67 HV. The results of the EIS test showed that the polarization resistance of the coating was at its maximum at this time, which could provide long-term effective protection for E235 steel.

## Figures and Tables

**Table 1:** Chemical composition of aluminum and zinc powder (wt.%).

	Si	Fe	Cu	Al	Cd	Pb	Zn
Al	0.03	0.058	0.001	Bal.	-	-	-
Zn	-	0.006	-	-	0.004	0.004	Bal.

**Table 2:** Chemical composition of substrate (wt.%).

	C	Mn	Si	S	P	Fe
E235	0.15	0.5	0.2	0.2	0.03	Bal.

**Table 3:** Optimization experiment of temperature parameters of cold spraying.

Samples	Spraying Distance (mm)	Temperature (°C)	Powder-Feeding Motor Speed (r/min)
1	20	550	1.5
2	20	500	1.5
3	20	450	1.5
4	20	400	1.5
5	18	500	1.5
6	20	500	1.5
7	22	500	1.5
8	25	500	1.5
9	27	500	1.5
10	30	500	1.5
11	27	500	1.0
12	27	500	1.2
13	27	500	1.5
14	27	500	1.7
15	27	500	2.0

**Table 4:** EIS fitting results of Al–Zn composite coating at different spraying temperatures in 3.5 wt % NaCl solution at the initial immersion stage.

	400 °C	450 °C	500 °C	550 °C
R_s_ (Ω)	14.4	14.1	17.5	15.7
R_e_ (Ω)	18,923	27,662	113,807	33,092

**Table 5:** EIS fitting results of Al–Zn composite coating at different spraying distances at the initial immersion stage in 3.5 wt % NaCl solution.

	18 mm	20 mm	22 mm	25 mm	27 mm	30 mm
R_s_ (Ω)	13.2	17.5	16.3	12.0	12.4	16.3
R_e_ (Ω)	30,177	113,807	63,820	168,360	196,320	61,621

**Table 6:** EIS fitting fruit of Al–Zn composite coating soaked in 3.5 wt % NaCl solution at the initial stage at different feeding motor speeds.

	1.0 r/min	1.2 r/min	1.5 r/min	1.7 r/min	2.0 r/min
R_s_ (Ω)	15.0	18.7	12.4	17.3	25.6
R_e_ (Ω)	43,996	75,702	196,320	89,694	79,047

## Data Availability

Not applicable.
